# Tracing Marine Algal and Terrestrial Plant Inputs During Cenozoic Marine Incursions in the Northern Central Myanmar Basin: A Biomarker Perspective

**DOI:** 10.3390/biology15110828

**Published:** 2026-05-25

**Authors:** Zengyuan Zhou, Yubo Shi, Tianhao Yan, Xianfeng Wang

**Affiliations:** 1 State Key Laboratory of Marine Geology, Tongji University, Shanghai 200092, China; 2 Key Laboratory of Deep-Time Geography and Environment Reconstruction and Applications of Ministry of Natural Resources, Chengdu University of Technology, Chengdu 610059, China; 3 Research Center for Marine Resources, Tongji University, Shanghai 200092, China; 4 Key Laboratory of Submarine Geosciences and Prospecting Technology, College of Marine Geosciences, Ocean University of China, Qingdao 266100, China

**Keywords:** biomarker, marine algal, terrestrial plant inputs, paleo-environment

## Abstract

Marine incursions can profoundly alter biological input and environmental conditions in transitional sedimentary basins, yet their ecological effects remain insufficiently understood in the northern Central Myanmar Basin (CMB). Here, we investigate Upper Cretaceous to Eocene mudrocks from the northern CMB using integrated organic biomarker and elemental geochemical analyses to reconstruct biological precursors, depositional environments, and ecosystem responses during seawater incursions. The biomarker assemblages, including n-alkanes, isoprenoids, tricyclic terpanes, and C_27_–C_29_ regular steranes, indicate persistent mixed inputs of marine algal organic matter and terrestrial higher-plant debris. In particular, the upward increase in C_29_ steranes from the Upper Cretaceous to the Eocene suggests a progressive strengthening of terrestrial input through time. Elemental proxies, including Sr/Ba, Th/U, Y/Ho, (Zn + Ni)/(Ga × 5), Sr/Cu, Rb/Sr, and V/(V + Ni), indicate that deposition occurred under marine-influenced, brackish to locally saline, warm–humid, and predominantly weakly reducing to reducing conditions. We interpret these patterns as evidence that marine incursions reorganized habitat conditions and biological input in a near-equatorial transitional ecosystem. The increasing contribution of terrestrial biomass was likely linked to the progressive uplift and exhumation of the Indo-Burman Ranges, which expanded exposed land area and enhanced the supply of land-derived organic matter to the basin. These results provide a biomarker-based perspective on how marine incursions and paleogeographic reorganization jointly shaped ecosystem dynamics and organic-matter preservation in the northern CMB.

## Simple Summary

Ancient coastal basins were shaped not only by changes in sea level, but also by the way marine waters entered continental environments and altered local ecosystems. In the northern Central Myanmar Basin, such marine incursions occurred from the Late Cretaceous to the Eocene. In this study, we used organic biomarkers preserved in mudrocks together with elemental geochemistry to investigate how these events affected biological input and environmental conditions. The results show that the basin received a persistent mixture of marine algal organic matter and terrestrial plant debris. At the same time, seawater incursions created brackish, suboxic to anoxic conditions that favored the preservation of mixed organic biomass. Through time, terrestrial plant-derived input became relatively more important, likely in response to the progressive uplift and emergence of the Indo-Burman Ranges, which expanded subaerial land area and enhanced the delivery of land-derived organic matter into the basin. These findings show that marine incursions in the northern Central Myanmar Basin were not only hydrological events, but also ecological disturbances that reorganized biological input in a near-equatorial transitional ecosystem.

## 1. Introduction

Reconstructing ancient ecosystems and understanding how biological communities responded to drastic environmental shifts are central to the field of paleoecology [1,2]. In geological records where the preservation of macroscopic plant or animal fossils is poor or biased, organic biomarkers (molecular fossils) serve as indispensable tools [3,4,5]. Specifically, lipid biomarkers such as regular steranes and tricyclic terpanes, preserved in sedimentary rocks, retain the structural skeletons of their biological precursors [6,7]. These molecular signatures allow researchers to accurately identify and quantify the diverse biological inputs—ranging from aquatic planktonic algae to terrestrial higher plants—and to trace the ecological succession driven by climatic or paleogeographic changes [8,9].

During the Late Cretaceous to the Eocene, the Central Myanmar Basin (CMB) occupied a near-equatorial position and harbored a highly dynamic and diverse transitional ecosystem. Recent palynological studies have provided crucial insights into the paleo-vegetation of this region. For instance, Huang et al. (2020, 2021, 2023) demonstrated that the Eocene CMB was characterized by highly diverse lowland evergreen forests, swampy coastal settings, and mangrove ecosystems, deeply influenced by a proto-monsoonal climate with distinct wet and dry seasons [10,11,12].

While macroscopic and palynological evidence has successfully painted a picture of the terrestrial flora and mangrove dynamics during these periods, a significant gap remains regarding the aquatic microbial community and the precise physicochemical conditions of the water column. Spores and pollen effectively trace terra firma and coastal vegetation [13,14,15,16], but they often overlook the influx of marine aquatic organisms (e.g., planktonic algae) that arrive with the invading seawater. Furthermore, understanding the exact nature of the habitat—such as paleo-salinity, redox conditions, and weathering intensity—is critical to explaining how marine and terrestrial biomass mixed and preserved within this shifting environment [17,18].

This study analyzed organic biomarkers and trace elements from the Upper Cretaceous to Eocene sediments in the northern CMB. Our main objectives are to identify the mixed biological inputs of marine algae and terrestrial plants, track ecological successions during marine incursions and reconstruct detailed paleohabitat conditions, including salinity, climate, and redox states. Ultimately, this study clarifies the dynamic response between ancient environments and biological communities, providing valuable insights into the ecological drivers of regional organic resource accumulation.

## 2. Materials and Methods

**Figure 1:**
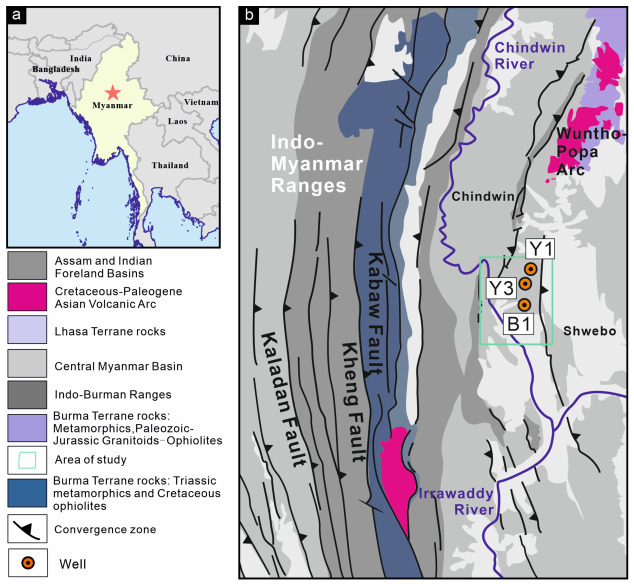
Geological map of Myanmar. (**a**) Star indicates location of study area; (**b**) Location of studied wells.

A total of sixty-three mudrock samples from the Upper Cretaceous to Eocene succession in the northern Central Myanmar Basin were collected for bulk-rock geochemical analysis, including published data from the adjacent Y1 well [19], and seven representative samples were selected for organic biomarker analysis (Figure 1). The biomarker samples were chosen to cover the main stratigraphic intervals of the Upper Cretaceous, Paleocene, and Eocene successions. First, we focused on lithological homogeneity and potential organic richness, specifically targeting dark, fine-grained mudrocks that favor the preservation of primary organic matter. Second, samples were meticulously screened to ensure the absence of visible weathering, fracturing, or secondary alteration that could compromise the lipid signatures. Finally, these selected samples were strategically spaced to cover the main stratigraphic intervals of the Upper Cretaceous, Paleocene, and Eocene successions, thereby ensuring a representative temporal coverage of the basin’s evolution. The data results are shown in Table 1.

### 2.1. Extraction and GC-MS Analysis of Organic Biomarkers

The analysis of saturated hydrocarbons provides direct molecular evidence of the biological precursors (e.g., marine algae versus terrestrial higher plants) contributing to the sedimentary organic matter. The sample preparation and extraction followed standard organic geochemistry protocols. First, the mudstone samples were pulverized, and the soluble organic matter was extracted using the traditional Soxhlet extraction method. The asphaltenes in the extract were then precipitated using n-hexane. Subsequently, the obtained filtrate was loaded onto a silica gel column. By sequentially applying solvents of increasing polarities, the extract was fractionated into saturated hydrocarbons, aromatic hydrocarbons, and non-hydrocarbon components [20,21,22].

The isolated saturated hydrocarbon fraction was analyzed using a Thermo Scientific Trace GC Ultra-DSQ GC-MS (Thermo Fisher Scientific, Waltham, MA, USA) equipped with an HP-5MS elastic quartz capillary column (60 m length × 0.25 mm inner diameter × 0.25 μm film thickness). The GC oven temperature program was initiated at 100 °C (held for 5 min), increased at a rate of 3 °C/min to 320 °C, and held at 320 °C for 20 min. High-purity helium (99.99%) was used as the carrier gas at a constant flow rate of 1 mL/min. The inlet temperature was set at 280 °C, and the transfer line was maintained at 300 °C. The mass spectrometer was operated in electron impact (EI) ionization mode at an electron energy of 70 eV, with a filament current of 100 mA and an ion source temperature of 250 °C. Biomarker distributions were identified from total ion chromatograms and selected ion chromatograms. N-alkanes and isoprenoids were used to evaluate the relative contributions of different organic matter sources, whereas tricyclic terpanes and regular steranes were used to characterize biological input from marine algal and terrestrial higher-plant sources [23,24].

### 2.2. Trace Element Analysis

Inorganic elemental analysis was conducted to quantify specific geochemical proxies for reconstructing the paleohabitat during marine incursions. Specifically, we targeted element ratios indicative of paleo-salinity (e.g., Sr/Ba, Th/U, Y/Ho, and (Zn + Ni)/(Ga × 5)), paleo-climate, Sr/Cu, and Rb/Sr), and paleo-redox conditions (e.g., V/Ni and V/(V + Ni)). The whole-rock elemental analysis was performed at Nanjing FocuMS Technology Co., Ltd. (Nanjing, China). Briefly, the powdered samples were completely digested using a mixture of HNO_3_ and HF acids in high-pressure PTFE bombs. After thorough digestion and re-dissolution, the diluted samples were analyzed using an Agilent Technologies 7700× quadrupole Inductively Coupled Plasma Mass Spectrometer (ICP-MS, Tokyo, Japan) to accurately measure the concentrations of the targeted major and trace elements. For relevant element index data, are provided in full in Table S1 of the Supplementary documents.

## 3. Results

### 3.1. Organic Biomarker Characteristics

**Figure 2:**
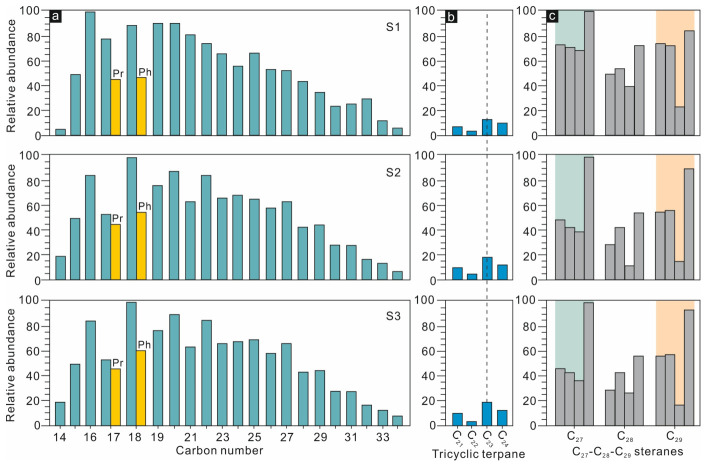
Molecular distribution of organic compounds in mudrock samples from well Y3 (S1 means Eocene, 1164 m depth; S2 means Paleocene, 1648 m depth; S3 means Upper Cretaceous, 2198 m depth). (**a**) Saturated hydrocarbon-gas chromatogram, highlighting the distribution of n-alkanes and isoprenoids (Pr: pristane; Ph: phytane); (**b**) Terpane *m*/*z* 191 mass chromatogram fragment; (**c**) Sterane *m*/*z* 217 mass chromatogram fragment.

The composition of n-alkanes and isoprenoid hydrocarbons provide information both on their bio-precursors and on depositional environments [25,26]. The distribution of N-alkanes in Upper Cretaceous to Eocene mudrocks is complete and dominated by low-carbon-number n-alkanes (Figure 2a). Carbon numbers range from C_16_ to C_31_ with bimodal distribution. The main peak of n-alkanes is C_18_ in the Upper Cretaceous to Paleocene, C_16_ in the Eocene. Due to the fact that pristane is an oxidative environmental product, while phytane is a reducing environmental product [27]. The Pr/Ph ratio is mainly <1.0; Pr/nC_17_ ranges from 0.37 to 1.96 (average 1.08) and Ph/nC_18_ from 0.23 to 0.86 (average 0.53). The distribution of tricyclic terpanes in the rocks is complete. The C_21_-C_24_ tricyclic terpanes, indicative of reducing environments, show maximum and minimum abundances for C_23_ and C_22_, respectively (Figure 2b). The regular steranes exhibit a typical “V” distribution (Figure 2c), with similar abundance for C_29_ and C_28_, whereas C_27_ occurs less commonly. The proportion of C_29_ gradually increases from the Upper Cretaceous to the Eocene.

### 3.2. Elemental Geochemistry

Trace-element ratios (e.g., Sr/Ba, Th/U, V/Ni) have been widely used to assess paleo-productivity and paleo-redox conditions during marine incursions [28,29,30], even though they are influenced by several other factors as well [31,32,33,34]. In samples from well B1, Sr/Ba mostly ranges between 0.2 and 0.4, with a few values exceeding 0.5. In samples from well Y3, instead, Sr/Ba mostly exceeds 0.5 and mainly ranges between 0.8 and 1.2. In all samples, Th/U mainly ranges between 3.0 and 5.0 and (Zn + Ni)/(Ga × 5) mainly between 1.0 and 2.0 but increasing to more than 2.0 in the mid-Paleocene. Y/Ho is mainly between 25 and 30, but only ~10 in a few samples.

The Sr/Cu and Rb/Sr ratios have also been proposed as weathering indicators of chemical [35,36,37]. In our samples, Sr/Cu ranges from 0 to 5 and Rb/Sr from 0 to 1, being >0.2 in ~80% of the samples. The V/(V + Ni) ratio, used to reflect reducing conditions, varies between 0.4 and 1.0, being >0.6 in 95% of the samples and ranging between 0.7 and 0.9 in most.

## 4. Discussion

### 4.1. Biological Precursors and the Marine-Terrestrial Mix

The biomarker assemblages indicate that the Upper Cretaceous to Eocene mudrocks of the northern Central Myanmar Basin presented a persistent mixture of marine and terrestrial organic matter. The n-alkane distributions are complete and dominated by low-carbon-number compounds [38,39,40], with carbon numbers ranging from C_16_ to C_31_ and showing a bimodal pattern (Figure 2a). The predominance of C_18_ in the Upper Cretaceous–Paleocene samples and the shift to C_16_ in the Eocene suggest that short-chain organic matter remained an important component throughout deposition, pointing to a substantial contribution from aquatic primary producers (Figure 2a). At the same time, the persistence of higher-carbon-number compounds indicates that terrigenous higher-plant debris was continuously supplied to the basin.

The terpane and sterane distributions further support this mixed-input interpretation. The complete distribution of C_21_–C_24_ tricyclic terpanes, with C_23_ as the dominant homolog and C_22_ as the least abundant (Figure 2b), is consistent with deposition under marine-influenced reducing conditions rather than in a purely continental oxidizing setting. Likewise, the regular steranes display a characteristic V-shaped pattern, with similar abundances of C_28_ and C_29_ and relatively lower C_27_ contents (Figure 2c). Such a distribution does not fit a simple end-member source model, but instead suggests coexistence of marine algal organic matter and terrestrial higher-plant debris in a marginal to restricted depositional environment. This interpretation is consistent with the overall conclusion of the study that the relative proportions of regular steranes and tricyclic terpanes point to mixed marine algal and terrestrial higher-plant inputs.

**Figure 3:**
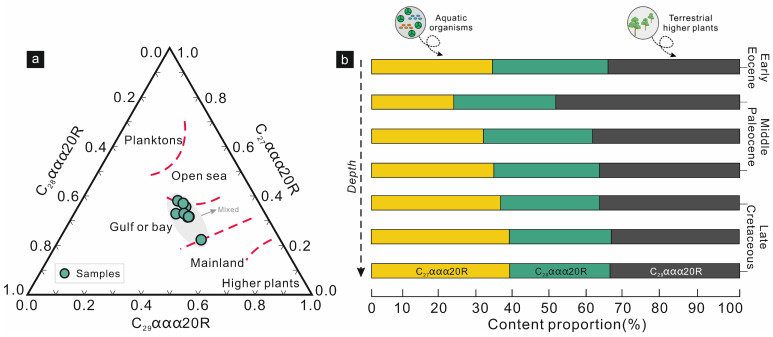
Ternary diagram showing the composition of C_27_–C_28_–C_29_ steranes in mudrock samples from the northern CMB. (**a**) Ternary diagram of C_27_–C_28_–C_29_ regular steranes; (**b**) Stratigraphic variation in the relative proportions of C_27_–C_28_–C_29_ steranes from the Late Cretaceous to the Early Eocene.

An additional stratigraphic feature is the upward increase in C_29_ steranes from the Upper Cretaceous to the Eocene (Figure 3). However, it is noteworthy that this long-term trend exhibits a localized fluctuation, specifically a temporary decrease in C_29_ steranes from the Middle Paleocene to the Early Eocene (Figure 3b). We interpret this short-term variation as a reflection of local depositional facies shifts within the highly heterogeneous transitional ecosystem. While the overarching basin evolution favored progressive terrestrialization, the CMB functioned as a complex mosaic of coastal plains, restricted lakes, and bays. During the Middle Paleocene to Early Eocene, the specific sedimentary environments at our sampling sites likely shifted toward more restricted aquatic sub-environments. In the ternary sterane diagram, most samples fall within the gulf or bay fields (Figure 3a), indicating persistent marine influence, but the simultaneous decrease in C_27_ and increase in C_29_ imply that the relative contribution of terrestrial higher-plant organic matter became progressively more important upward through the succession. Rather than indicating a complete replacement of marine input by land-derived organic matter, this trend more likely reflects changing proportions within a long-lived transitional system, in which marine incursions were superimposed on continuous terrigenous supply.

### 4.2. Paleoenvironmental Reconstruction

#### 4.2.1. Marine Incursions and Paleo-Salinity

**Figure 4:**
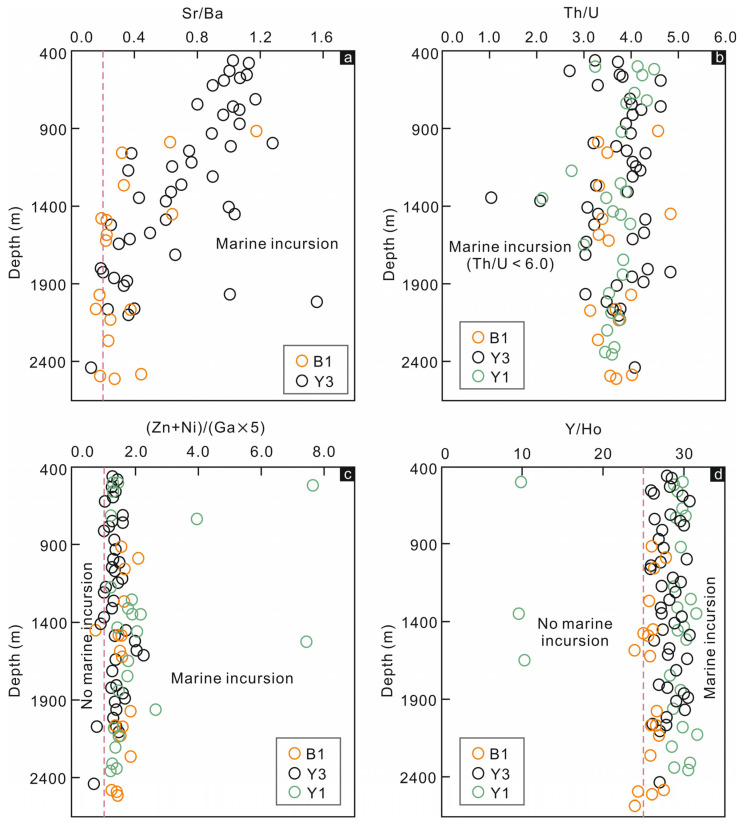
Trace-element evidence for marine incursions in the northern CMB. (**a**) Sr/Ba *versus* depth; (**b**) Th/U *versus* depth; (**c**) (Zn + Ni)/(Ga × 5) *versus* depth; (**d**) Y/Ho *versus* depth. (Note: For clarity of visualization, three data points with values (672, 28.2), (921, 12.3) and (1346, 15.1) are omitted from (**c**). These points represent valid observations that are consistent with the overall trend of the dataset.)

Trace elements and biomarker compounds provide precious information on the evolution of depositional environments during marine incursions in the CMB (Table 2). Because Sr has a much longer residence time in seawater (~2.4 Ma) than Ba (11 ka) [41,42], an increase in salinity levels can be revealed by increasing Sr/Ba ratio in sediments [42,43]. Sr/Ba ratios of <0.2, 0.2–0.5, and >0.5 would thus be indicative of freshwater, brackish water, and marine environments, respectively. A Th/U ratio < 6 has been considered as an indicator of marine incursions [44]. The surface complexation of Y and Ho differs in seawater [45,46]. Ho precipitates twice as fast as Y as water depth increases, resulting in higher Y/Ho ratio in seawater than in freshwater. The (Zn + Ni)/(Ga × 5), Th/U, Sr/Ba, and Y/Ho values in the studied samples suggest that more than 90% of them were affected by marine incursions (Figure 4).

#### 4.2.2. Paleo-Climate

**Figure 5:**
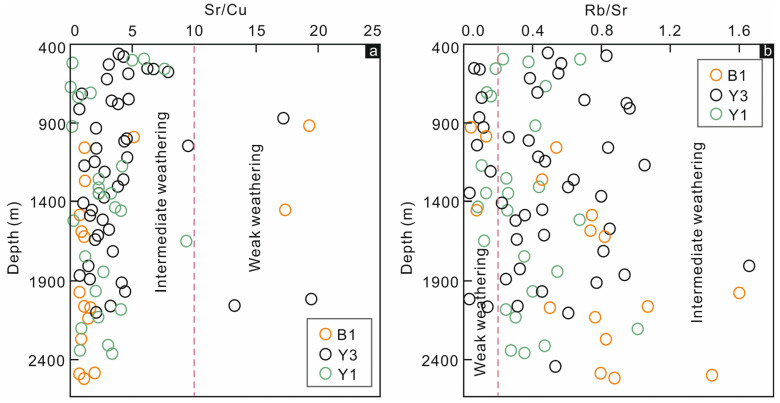
Rb/Sr and Sr/Cu ratios in northern CMB samples consistently indicate significant weathering, slightly decreasing in the Eocene. (**a**) Sr/Cu *versus* depth; (**b**) Rb/Sr *versus* depth. (Note: For clarity of visualization, one data point with values (2440, 89.8) was omitted from (**a**). This point represents valid observations that are consistent with the overall trend of the dataset).

Geochemical proxies and climate-sensitive trace elements (e.g., Rb, Sr, Cu) have been widely used as tools to reconstruct paleo-climate conditions [50]. The Upper Cretaceous to Eocene clastic rocks have low maturity and are mainly sourced from nearby local materials [51]. Sr and Cu are hygrometric elements and Sr/Cu ratios < 10 and >10 have been considered as proxies of humid and dry–hot climate, respectively. The Rb/Sr ratio has been shown to increase with higher mean annual temperature and precipitation [52]. Considering its near-equatorial paleo-latitude since the late Eocene, the CMB has been envisaged as covered by evergreen forests and swamps [10,53]. Proximity to the sea and increased precipitation due to marine moisture would have led to intense weathering in hot–humid climate (Figure 5a,b).

#### 4.2.3. Paleo-Redox Conditions

**Figure 6:**
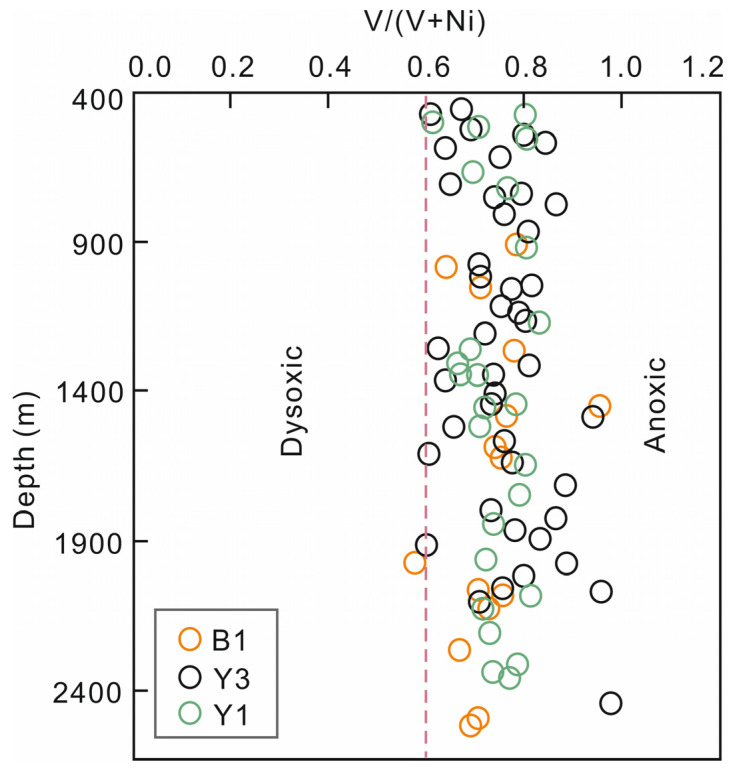
V/(V + Ni) values depth in the three studied cores in the northern CMB.

**Figure 7:**
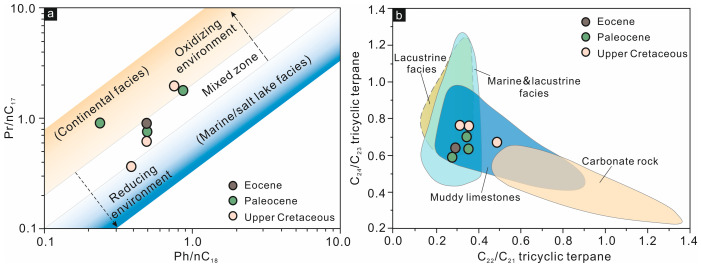
Biomarker compounds indicate reducing paleo-environments during marine incursions. (**a**) Pr/nC_17_-Pr/nC_18_ crossplot [59]; (**b**) C_24_/C_23_ *versus* C_22_/C_21_ tricyclic terpanes [60].

Trace element indicators such as V/(V + Ni) [31] and biomarker ratios of pristane–adjacent n-alkane (Pr/nC_17_), phytane–adjacent n-alkane (Ph/nC_18_), and C_21_-C_24_ tricyclic terpanes are widely used to indicate redox conditions [54,55,56,57,58]. In reducing anoxic environments, V/(V + Ni) is typically >0.84, whereas it ranges from 0.60 to 0.84 in weakly anoxic sub-reducing conditions with less pronounced water column stratification, and is <0.60 in oxic environments. In most of our samples, V/(V + Ni) is >0.6 (Figure 6), suggesting sub-reducing conditions. Biomarker ratios of isoprenoid hydrocarbons (Pr/nC_17_ and Ph/nC_18_) independently suggest reducing to weakly reducing conditions (Figure 7a), and the C_24_/C_23_ versus C_22_/C_21_ plot indicates reducing environments in both marine and lacustrine settings (Figure 7b).

**Figure 8:**
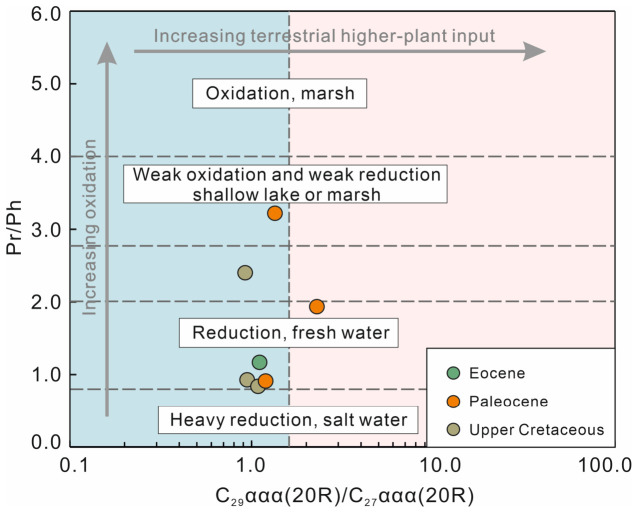
Cross-plot of C_29_/C_27_ααα(20R) regular steranes versus Pr/Ph ratios for mudrock samples from the northern CMB.

A cross-plot of C_29_/C_27_ααα(20R) steranes versus Pr/Ph was utilized (Figure 8). The distribution of samples lacks the extreme end-member signatures of pure terrestrial oxidation (high Pr/Ph and high C_29_/C_27_) or pure marine algal input. Instead, the samples cluster in an intermediate position characterized by Pr/Ph ratios indicative of weakly reducing to reducing conditions and intermediate C_29_/C_27_ ratios. This specific covariant clustering indicates a mixed origin for the organic matter. Rather than being exclusively terrigenous, the C_29_/C_27_ steranes here likely reflect a combination of terrestrial higher-plant debris and significant aquatic algal inputs. This mixed biomarker assemblage perfectly corroborates our elemental geochemical findings, which point to a dynamic, transitional gulf or bay paleoenvironment subjected to recurrent marine incursions.

### 4.3. Ecological Response and Organic Biomass Preservation

When the biomarker and geochemical evidence are considered together, it becomes clear that marine incursions did not simply alter the water chemistry of the northern CMB, they also reorganized the structure of biological input within a dynamic transitional ecosystem. Seawater incursions periodically shifted the basin away from more freshwater-influenced conditions toward brackish to locally saline settings. Under these circumstances, marine-derived organic matter, including algal input, was introduced into a sedimentary system that continued to receive abundant terrestrial higher-plant debris from adjacent land areas. The resulting assemblage was therefore not dominated by a single ecological source, but instead presented a persistent marine–terrestrial organic mixture.

This mixed-input character has important paleo-ecological implications. It suggests that the northern CMB functioned as a near-equatorial coastal to marginal-marine mosaic, in which terrestrial vegetation, swamp-derived plant debris, and marine organic matter coexisted within the same depositional system. In such a setting, marine incursions acted as recurrent ecological disturbances: they modified salinity and water-column chemistry, changed the balance between marine and terrestrial biological precursors, and restructured the environments in which organic matter accumulated.

**Figure 9:**
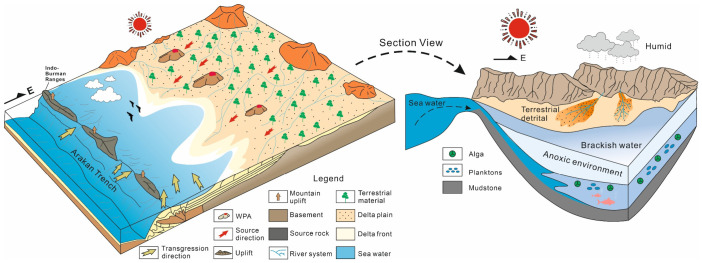
Envisaged scenario of marine incursions into the eastern CMB, leading to deposition of mixed marine algal and terrestrial plant debris derived from southern Andaman Sea and western Bengal Sea regions.

Crucially, the upward increase in C_29_ steranes implies that the balance of biological input shifted through time, with terrestrial higher-plant contributions becoming relatively stronger. This temporal ecological shift was fundamentally driven by the regional paleogeographic reorganization associated with the Indo-Burman Ranges (IBR). During the Late Cretaceous to Paleocene, the nascent IBR was primarily an underwater accretionary prism, allowing the CMB to maintain an open connection to the proto-Bay of Bengal [61,62,63,64]. However, driven by the ongoing collision and accretion within the Tethyan domain, the IBR experienced accelerated exhumation and subaerial emergence starting in the early-middle Eocene. From an ecological perspective, this tectonic uplift had a dual effect: it gradually restricted the western marine invasion pathways, and, more importantly, it significantly expanded the exposed subaerial landmass. The newly emerged topography provided extensive new habitats for the colonization of terrestrial and coastal flora. Consequently, the intensified riverine runoff from these newly vegetated highlands delivered increasingly abundant terrestrial plant debris into the basin (Figure 9). This dynamic is perfectly captured in the C_27_-C_28_-C_29_ sterane ternary diagram, where samples primarily plot in the gulf or bay fields, yet display a long-term stratigraphic trend of decreasing C_27_αααR (marine algae) and increasing C_29_αααR (terrestrial plants) content, mirroring the strengthening of the IBR and the progressive terrestrialization of the basin margins.

The depositional environment also directly controlled the preservation of this mixed biomass. Weakly reducing to reducing conditions, as indicated by both V/(V + Ni) and biomarker parameters, would have favored the retention of marine and terrestrial organic matter in muddy sediments. This means that marine incursions—whether flowing from the southern Andaman Sea or leaking through remaining northern gaps in the IBR—were not only hydrological events but also preservational windows; they introduced marine organic matter into the basin while simultaneously establishing stratified, oxygen-depleted conditions under which that signal could be retained (Figure 9). In this sense, the northern CMB can be interpreted as a transitional paleo-ecosystem in which marine incursions and concurrent topographic uplift reshaped habitat conditions, altered the relative importance of marine and terrestrial biological inputs, and ultimately enhanced the preservation of mixed organic biomass, establishing the material foundation for the region’s hydrocarbon potential.

## 5. Conclusions

This study integrates organic biomarker and elemental geochemical evidence to investigate biological input and paleoenvironmental change during Late Cretaceous to Eocene marine incursions in the northern Central Myanmar Basin. The main conclusions are as follows:

Organic biomarker assemblages indicate persistent mixed inputs of marine algal organic matter and terrestrial higher-plant debris throughout the studied succession. The n-alkane, tricyclic terpane, and regular sterane distributions collectively show that the northern CMB functioned as a long-lived marine–terrestrial transitional system. Elemental geochemical proxies indicate that deposition took place under marine influence, with predominantly brackish to locally saline waters, a generally warm–humid climatic–hydrological background, and weakly reducing to reducing depositional conditions. The upward increase in C_29_ steranes from the Upper Cretaceous to the Eocene suggests that terrestrial organic input became progressively more important through time. This trend is interpreted to reflect the progressive uplift and exhumation of the Indo-Burman Ranges, which expanded exposed land area and enhanced the supply of land-derived plant debris to the basin.

Marine incursions in the northern CMB were therefore not only hydrological events, but also ecological disturbances that reshaped habitat conditions, reorganized biological input, and promoted the preservation of mixed organic biomass in a near-equatorial transitional paleo-ecosystem.

## Figures and Tables

**Table 1:** Summary of indicator parameters of biomarker compounds in mudstone extracts.

Sample No	Lithology	Elevation/ Depth (m)	Geological Time	Ph/nC_18_	Pr/nC_17_	C_27_αααR	C_28_αααR	C_29_αααR	C_22_/C_21_	C_24_/C_23_
1	dark mudstone	1169	Eocene	0.49	0.76	0.33	0.31	0.36	0.27	0.65
2	dark mudstone	1690	Paleocene	0.48	0.90	0.22	0.28	0.50	0.26	0.62
3	dark mudstone	1725	Paleocene	0.23	0.91	0.31	0.28	0.41	0.29	0.65
4	dark mudstone	1896	Paleocene	0.86	1.78	0.33	0.29	0.38	0.34	0.72
5	dark mudstone	2023	Late Cretaceous	0.42	0.37	0.35	0.27	0.38	0.50	0.68
6	dark mudstone	2106	Late Cretaceous	0.48	0.62	0.37	0.27	0.35	0.37	0.76
7	dark mudstone	2182	Late Cretaceous	0.74	1.96	0.38	0.28	0.35	0.32	0.75

**Table 2:** Criteria for identification of marine incursions in the northern CMB (- = not applicable).

Indicator	Marine Incursion	Little or No Marine Incursion	References	Well Y1 (Marine Incursion)	Well Y3 (Marine Incursion)	Well B1 (Marine Incursion)
Th/U	<6.0	>6.0	Degens, 1965 [47]	100%	100%	94%
(Zn + Ni)/(Ga × 5)	>1–1.5	-	Wang et al., 2002 [48]	100%	100%	100%
Y/Ho	>25	<25	Tostevin et al., 2016 [49]	88%	87%	67%
Sr/Ba	>0.20	<0.20	Wei and Algeo, 2020 [42]	-	89%	78%

## Data Availability

The original contributions presented in the study are included in the article, further inquiries can be directed to the corresponding authors.

## Supplementary Materials

The following supporting information can be downloaded at: https://www.mdpi.com/article/10.3390/biology15110828/s1, Table S1: Trace element geochemical ratio data of Upper Cretaceous-Paleocene and Eocene drillings in the northern Central Myanmar Basin.
